# Risk of preterm birth and second-trimester loss following intrapartum cesarean sections and uterine extensions: a population-based cohort study

**DOI:** 10.1186/s12978-026-02387-w

**Published:** 2026-06-19

**Authors:** Fathima Minisha, Jis Thomas, Feazlin Din, Sadia Mehmood, Dhannya Stanley John, Asma Gul, Salwa Abu Yaqoub, Zeena Bu Shurbak, Nader Aldewik, Megan Hall, Andrew Shennan, Thomas Farrell

**Affiliations:** 1 Department of Obstetrics and Gynaecology, Women’s Wellness and Research Centre, Hamad Medical Corporation, Doha, Qatar; 2 Research Centre, Women’s Wellness and Research Centre, Hamad Medical Corporation, Doha, Qatar; 3 College of Medicine, Qatar University, Doha, Qatar; 4 Department of Women and Children’s Health, King’s College London, London, UK

**Keywords:** Cesarean section at full dilatation, CSFD, Preterm birth, Second-trimester miscarriage, Uterine incision extension

## Abstract

**Background:**

Preterm birth (PTB) is associated with significant neonatal morbidity and mortality. Antenatal risk assessment is crucial for prevention and management. Recent evidence suggests that cervical injury during cesarean is a potential risk factor. This study aimed to evaluate the impact of intrapartum cesarean, specifically at full cervical dilatation (CSFD), on subsequent second-trimester loss and preterm birth.

**Methods:**

Women delivering by their first term cesarean in 2017 were included in this retrospective cohort study, and data on all subsequent pregnancies till 2024 were extracted. They were divided based on the type of cesarean- elective (CS-E) versus cesarean in latent labour (CS-L), cesarean in active labour (CS-A) and CSFD (± uterine extensions). Adjusted risk ratios (aRR) with 95% confidence intervals (CIs) were calculated to evaluate associations with subsequent pregnancy outcomes.

**Results:**

Among 984 women, 267 had CS-E, 256 had CS-L, 212 had CS-A and 249 had CSFD. The incidence of spontaneous PTB < 37 weeks was 17.9% in CSFD (3–6% in others; *p* < 0.001). 26.7% of those who had a cesarean for failed instrumental delivery (FID) had a subsequent sPTB. 10.7% (vs. < 1%) and 12.5% (vs. < 6%) of CSFD with 3–5 cm extension had a second-trimester miscarriage and sPTB < 34 weeks, respectively. CSFD group had a 6.5 times higher risk of sPTB < 37 weeks compared to CS-E (CI 2.47–16.9; *p* < 0.001); the risk increased to 8.2 times in CSFD with 3–5 cm extensions (CI 2.27–29.5, *p* = 0.001). For every cm increase in cervical dilatation at the time of the index cesarean, the risk increased by 27% (CI 1.14–1.41;*p* < 0.001). Cesarean for no descent of head and FID had 5.3 times (CI 2.20–12.8; *p* < 0.001) and 8.7 times higher risk (CI 2.72-28.0; *p* < 0.001), respectively.

**Conclusion:**

CSFD is associated with a 6.5-fold increase in risk of spontaneous preterm birth. Uterine extension was an important predictor for second-trimester loss and sPTB. Risk assessment for sPTB should potentially include detailed cesarean history, and women with CSFD should potentially be referred to dedicated PTB clinics and offered preventive measures such as serial cervical length assessments, vaginal progesterone and cervical cerclage.

**Supplementary Information:**

The online version contains supplementary material available at https://doi.org/10.1186/s12978-026-02387-w.

## Background

Preterm birth (PTB), a persistent challenge in obstetrics, is associated with significant neonatal and childhood morbidity and healthcare costs, ultimately leading to negative psychological and socioeconomic consequences for the parents [1]. Approximately 13–14 million babies are born preterm annually [2], with the rates remaining consistent for decades. The development of risk assessment strategies, preventive and predictive methods, is taking top priority in most units worldwide. Cervical injury and subsequent loss of integrity and function, resulting from surgical procedures or previous traumatic deliveries, are a major risk factor for PTB and second-trimester miscarriages [3].

The rates of cesarean sections (CS) have risen worldwide, driven by repeat cesareans, and the rising influence of patient choices on decision-making [4]. There is an increasing trend in the rates of cesarean sections at full dilatation (CSFD) as well, the reasons being multifactorial, such as decreased attempts at instrumental deliveries due to concerns about safety and litigation, and eventual loss of skills for advanced operative vaginal births [5]. Unlike other indications, CSFD is inherently more traumatic for the mother, technically more challenging and associated with significant maternal surgical morbidity and increased neonatal morbidity [4, 6]. 

There is a rising concern about subsequent pregnancies in those who have had CSFD. Approximately 1/4th will have uterine incision extensions that might include the cervix, and the majority inadvertently cause some form of cervical injury since the cervix is completely pulled up to the lower uterine segment at full dilatation [7]. The pressure of the fetal head makes the tissue fragile, highly vascular and swollen, increasing the chances of extensions [8]. Several studies globally show an increased risk of spontaneous preterm birth (sPTB) in those with prior CSFD [7, 9–11], compared to both CS in the first stage of labour and those who had a prior vaginal birth (VB). Currently, the Royal College of Obstetricians and Gynecologists (RCOG) guidelines recommend cervical length surveillance in those who have had a CSFD, considering it an intermediate risk factor for second-trimester pregnancy loss and sPTB [12]. 

Qatar has a PTB rate of 8–9%, with factors such as age, parity, comorbidities, nationality, and BMI reported as significant predictors [13]. However, the impact of previous CS on sPTB or second-trimester miscarriages has not been explored yet, representing a critical gap since more than one-third of deliveries in Qatar are by CS [14, 15], 40% of which are performed during labour (5% in the second-stage) as per local data. Current local practice does not recommend routine cervical length screening or cervical cerclage insertion in this cohort. This study attempted to bridge this gap in knowledge and clinical practice by comparing the incidence of subsequent pregnancy outcomes in those who had a CSFD or a CS in the first stage of labour (latent and active phase) with those who had an elective CS.

## Methods

### Study design and participants

A population-based retrospective cohort study, with an eight-year follow-up period, was conducted at the largest central tertiary care maternity hospital in Qatar, where nearly 1,400 births occur per month. Ethical approval was obtained from the Institutional Review Board of Hamad Medical Corporation (MRC-01-24-799), with a waiver of informed consent as only preexisting data from electronic medical records were used.

All women delivering singleton term babies (≥ 37 completed weeks), by cesarean between January and December 2017 at the tertiary centre, were considered for inclusion in this study (25% of all births). Women with previous cesarean births, multiple gestations, congenital anomalies and stillbirths were then excluded. They were divided into four groups based on the nature of their CS- planned elective cesarean (CS-E), cesarean in the latent phase of labour- CS-L (cervical dilatation 1–5 cm), cesarean in the active phase of labour- CS-A (cervix 6–9 cm) [16] and cesarean at full cervical dilatation- CSFD. Data regarding all subsequent pregnancies occurring between the index delivery in 2017 and December 2024 were extracted.

### Variables and data source

Data was extracted from electronic medical records by five experienced investigators and entered into a secure online database, with patient-identifying variables coded appropriately to preserve data anonymity. Double data entry and intermittent data quality checks were done to ensure accuracy Details about the index pregnancy in 2017 included maternal age in completed years, parity defined as number of previous births ≥ 24 weeks gestational age (GA), nationality as documented in national records, history of PTB or second-trimester miscarriage, preexisting comorbidities such as diabetes, hypertension, thyroid disorders or uterine fibroids, antenatal complications such as gestational diabetes (GDM), pregnancy induced hypertension (PIH), cholestasis of pregnancy, concerns with fetal growth including intrauterine growth restriction (IUGR) and large for date (LFD), and any placental concerns such as placenta previa.

Delivery details of the index pregnancy included GA at delivery in completed weeks, postdated pregnancy (≥ 41 weeks GA), indication for CS- such as abnormal lie or presentation, placenta previa, maternal comorbidity or request, no progress of labour or no descent of the head at full dilatation (NDH), suspected fetal distress or failed instrumental delivery (FID), estimated intraoperative blood loss (EBL) in millilitres, intraoperative complications such as hemorrhage (≥ 1000 ml EBL), presence of inadvertent extension of the uterine incision due to challenging delivery of impacted fetal head or extraction of the baby (including the degree of extension in centimetres) and any postoperative infection. The GA (in completed weeks) was determined according to a first-trimester dating scan or the last menstrual period (LMP) if the former was unavailable.

The interpregnancy intervals were calculated in months from the date of delivery till the LMP of the next subsequent pregnancy. For each subsequent pregnancy, the pregnancy outcomes considered were first-trimester miscarriage (loss of pregnancy until 14 weeks GA), second-trimester miscarriage (loss of pregnancy between 14 weeks and 23 weeks GA) and viable births- (delivery at ≥ 24 completed weeks GA). The definitions for the first and second trimester were based on our local clinical practise and the traditional threshold for first-trimester aneuploidy screening. PTB was defined as viable births < 37 weeks GA (incidence calculated as number of deliveries between 24 weeks till < 37 weeks divided by the total number of viable births > 24 weeks GA), and indications for PTB included iatrogenic (pregnancy terminated prematurely due to maternal or fetal risks), or spontaneous (sPTB)- defined as unexplained, spontaneous, preterm onset of uterine contractions, cervical dilatation and delivery with or without ruptured membranes [17]. sPTB < 34 weeks GA was evaluated separately due to increased neonatal morbidity associated with this group.

### Statistical analysis

Continuous variables were reported as means with standard deviations (SD) or medians with interquartile ranges (IQR), depending on the distribution of the variables. The distributions were evaluated visually using histograms and boxplots, supported by Shapiro-Wilk test. Means and medians were compared between the four exposure groups using one-way ANOVA and Kruskal-Wallis test, respectively. Categorical variables were represented as frequencies and percentages and compared using Chi-Square test or Fisher’s exact, if feasible.

The outcomes of the first subsequent pregnancy were evaluated among those who had a successful conception after the index pregnancy. The denominator for the delivery outcomes, such as PTB, was the number of births at ≥ 24 weeks. The risk was calculated as follows-

$$\:sPTB\:risk=\frac{Number\:of\:women\:having\:sPTB\:in\:their\:first\:subsequent\:conception\:(24w-36w)}{Number\:of\:women\:having\:a\:viable\:birth\:in\:the\:first\:subsequent\:conception\:(24w-42w)}$$

Risk ratios (RR) with 95% confidence intervals (CI) were calculated using generalised linear models for the binomial family (link-log) for the primary outcome- sPTB < 37 weeks (robust variance estimates). Apart from the main exposure groups (CS-E baseline), RRs were also determined for every unit increase in cervical dilatation at the time of index CS, indication for the index CS, and CSFD with extensions (none or ≤2 cm versus 3-5 cm). All associations were adjusted for potential confounders, including maternal age, BMI, parity, nationality, preexisting comorbidities and history of PTB or second-trimester loss, to obtain adjusted RRs. Collinearity was evaluated using variance inflation factors (VIF), aiming to exclude variables with high collinearity (VIF > 5). Since past obstetric history is an important predictor of subsequent sPTB, the analysis was repeated in women who were primigravida in 2017 (subgroup analysis). The predicted probabilities were plotted with 95% CI to demonstrate the trends. A two-sided p-value < 0.05 was considered strong evidence against the null hypothesis of no difference between exposure groups. All analyses were performed using Stata statistical software, Release 18, College Station, TX: Stata Corp LLC [18]. Descriptive analyses for all further conceptions over the follow-up period were performed as for the first conception.

The study was powered to compare the proportions of sPTB in CS-E and CSFD. For a risk ratio (RR) of at least 2.5, sPTB < 37 weeks rate of 8% in CS-E, alpha 0.05 and beta 0.2, the calculated sample size was at least 131 women with subsequent viable births in each group. We expected 50–60% of women to have a subsequent viable birth, resulting in 220–260 women being included per group.

## Results

### Index pregnancy (2017)

**Fig. 1:**
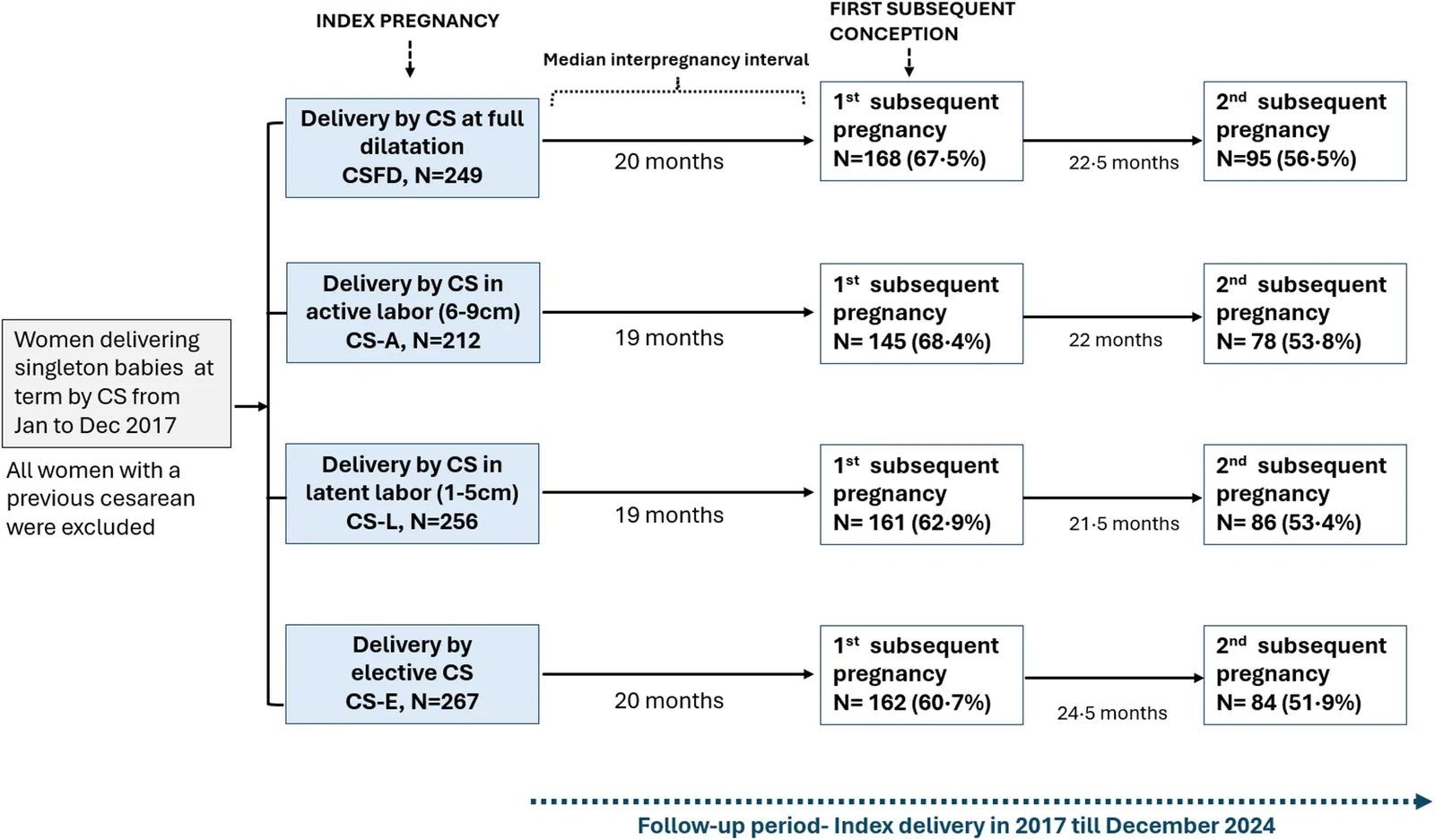
Study population; CS- Cesarean section

Among the 984 women included in the study (Fig. 1; Table 1), those with CS-A and CSFD were statistically significantly younger, with nearly 80% of CSFD being nulliparous. Nearly 44% of women having CS-E were Qatari, significantly higher than other groups. Fewer women with CSFD had preexisting medical illness or any pregnancy complications. Except in CS-E, 14–15% of the groups had a post-dated pregnancy. The indication was NDH in 70% of CSFD, with an additional 25 women (10%) having a cesarean after FID, whereas 60% of CS-L was done for suspected fetal compromise, as also evident by the higher proportion of fetal growth restriction in this group. Nearly 21% of CSFD had extensions (9% documented as 3–5 cm), compared to only 12.3% in CS-A and 3.5% in CS-L. More than 7% of CSFD and CS-L had post-operative infection, significantly higher than the other groups.

**Table 1:** Maternal, pregnancy and delivery characteristics in the index pregnancy in 2017

Maternal characteristics in Index pregnancy (2017)		Elective CS *N* = 267	CS in latent labour *N* = 256	CS in active labour *N* = 212	CS at full dilatation *N* = 249	*P* value
Maternal age in years (Mean, SD)		31.1 ± 6.3	29.6 ± 6.0	28.7 ± 5.8	28.4 ± 4.8	< 0.001
Parity (Median, IQR)		1 (0 to 2)	0 (0–2)	0 (0–1)	0 (0 to 0)	< 0.001
Nulliparity, n(%N)		137 (51.3%)	157 (61.3%)	138 (65.1%)	198 (79.5%)	< 0.001
History of PTB or 2nd-trimester miscarriage (n,%N)		12 (4.5%)	13 (5.1%)	7 (3.3%)	10 (4.0%)	0.809
Qatari nationality, n(%N)		116 (43.5%)	87 (34.0%)	62 (29.3%)	68 (27.3%)	< 0.001
Any preexisting medical illness, n(%N)		93 (34.8%)	48 (18.8%)	87 (41.0%)	42 (16.9%)	< 0.001
Uterine fibroids, (n, %N)		11 (4.1%)	2 (0.8%)	2 (0.9%)	0 (0%)	0.001
Gestational diabetes, n(%N)		58 (21.7%)	59 (23.1%)	51 (24.1%)	54 (21.7%)	0.914
Pregnancy induced hypertension, n(%N)		4 (1.5%)	23 (9.0%)	19 (9.0%)	15 (6.0%)	0.001
Cholestasis of pregnancy, n(%N)		6 (2.3%)	3 (1.2%)	2 (0.9%)	4 (1.6%)	0.688
Large for date fetus, n(%N)		14 (5.2%)	11 (4.3%)	12 (5.7%)	20 (8.0%)	0.322
Placenta previa, n(%N)		28 (10.5%)	2 (0.8%)	0 (0%)	0 (0%)	< 0.001
Any pregnancy complication, n(%N)		170 (63.7%)	160 (62.5%)	113 (53.3%)	126 (50.6%)	0.004
Gestational age at delivery (Median, IQR)		38 (38 to 39)	39 (38 to 40)	39 (39 to 40)	39 (39 to 40)	< 0.001
Post-dated pregnancy (≥ 41 weeks), n(%N)		4 (1.5%)	37 (14.5%)	30 (14.2%)	34 (13.7%)	< 0.001
Indication for cesarean, n(%N)	No descent of the head	0 (0.0%)	0 (0.0%)	0 (0.0%)	170 (68.3%)	
	No progress in labour	0 (0.0%)	45 (17.6%)	103 (48.6%)	0 (0.0%)	
	Suspected fetal distress	0 (0.0%)	154 (60.2%)	92 (45.5%)	49 (19.7%)	
	Failed instrumental delivery	0 (0.0%)	0 (0.0%)	0 (0.0%)	25 (10.0%)	
	Abnormal lie/ presentation	108 (40.4%)	49 (19.1%)	15 (7.1%)	3 (1.2%)	
	Placenta previa	29 (10.9%)	0 (0.0%)	0 (0%)	0 (0.0%)	
	Maternal comorbidity/ request	130 (48.7%)	7 (2.7%)	0 (0.0%)	0 (0.0%)	
Estimate blood loss in ml (Median, IQR)		400 (300–500)	400 (300–500)	400 (350–650)	500 (400–700)	< 0.001
Post partum hemorrhage (≥ 1000 ml), n(%N)		19 (7.1%)	9 (3.5%)	14 (6.6%)	16 (6.4%)	0.860
Uterine incision extensions, n(%N)	Nil	267 (100%)	247 (96.5%)	188 (88.7%)	198 (79.5%)	< 0.001
	Unilateral/ Bilateral/ Midline	0 (0%)	9 (3.5%)	26 (12.3%)	51 (20.5%)	
Degree of extension, n(%N)	Size not documented	0 (0%)	0 (0%)	1 (0.5%)	9 (3.7%)	< 0.001
	1–2 cm	0 (0%)	4 (1.6%)	13 (6.1%)	19 (7.6%)	
	3–5 cm	0 (0%)	5 (2.0%)	12 (5.7%)	23 (9.2%)	
Post operative infection, n(%N)		3 (1.1%)	19 (7.4%)	9 (4.3%)	18 (7.2%)	0.002

Means compared using one-way ANOVA, medians compared using Kruskal-Wallis test

Categorical variables compared using Chi-Square

*SD* standard deviation, *IQR* interquartile range, *PTB* preterm birth, *CS* Cesarean section

*p* < 0.05 considered statistically significant

### First subsequent conception

More than 60% in all groups had a subsequent conception (68% in CSFD and CS-L). The median interpregnancy interval for the first conception was 19–20 months across all groups (*p* = 0.442), the shortest interval being 2 months and the longest being nearly 8 years. Among these pregnancies, more than 80% were viable births, with 14% having first-trimester miscarriages and 1% having second-trimester miscarriages (Table 2).

**Table 2:** Outcomes of the first subsequent pregnancy after the index pregnancy

Outcomes of the first subsequent pregnancy *N*= number of first subsequent conception		Elective CS *N* = 162 (60.7%)	CS in latent labour *N* = 161 (62.9%)	CS in active labour *N* = 145 (68.4%)	CSFD *N* = 168 (67.5%)	*P* value
Months to first pregnancy (median, IQR)		20 (12–41)	19 (11–36)	19 (11–32)	20 (12-32.5)	0.442
Months to first pregnancy in Para 1 (median, IQR)		18 (11–36)	19 (11–29)	18 (10–32)	18 (12–30)	0.879
Pregnancy outcomes (7 missing) n(%N)	1st trimester loss	21 (13.0%)	19 (12.2%)	28 (20.1%)	22 (13.3%)	0.317
	2nd trimester loss	2 (1.2%)	2 (1.3%)	0 (0%)	3 (1.8%)	
	Viable births (> 23 weeks)	139 (85.8%)	135 (86.5%)	115 (80.0%)	141 (84.9%)	
Outcomes of the first subsequent viable pregnancy N = number of first viable pregnancy (Missing *N* = 13)		Elective CS *N* = 139	CS in latent labour *N* = 135	CS in active labour *N* = 115	CSFD *N* = 141	P value
Gestational age at delivery (Median IQR)		38 (38–39)	39 (38-39.5)	39 (38–39)	38 (37–39)	< 0.001
Rupture of membranes before birth, n(%N)		19 (14.0)	13 (10.3%)	17 (16.0%)	14 (10.0%)	0.416
Preterm birth < 34 weeks n(%N)		3 (2.2%)	0 (0%)	2 (1.8%)	11 (7.9%)	< 0.001
Preterm birth < 37 weeks n(%N)		10 (7.4%)	8 (6.2%)	8 (7.1%)	31 (22.1%)	< 0.001
Type of preterm < 37 weeks, n(%N)	Spontaneous preterm	5 (3.7%)	4 (3.1%)	7 (6.3%)	25 (17.9%)	< 0.001
	Iatrogenic preterm	5 (3.7%)	4 (3.1%)	1 (0.9%)	6 (4.3%)	
Mode of delivery n(%N)	Elective CS	77 (57.0%)	57 (44.5%)	46 (41.8%)	45 (32.6%)	0.002
	Emergency CS in labour	31 (23.0%)	44 (34.4%)	45 (40.9%)	61 (44.2%)	
	Successful VBAC	27 (20.0%)	27 (21.1%)	19 (17.3%)	32 (23.2%)	
Birthweight in grams (Mean, SD)		3157 ± 562	3206 ± 598	3145 ± 570	3170 ± 562	0.865
Admission to NICU n(%N)	Preterm	6 (60.0%)	4 (50.0%)	7 (87.5%)	21 (72.4%)	0.309
	Term	12 (10.3%)	8 (6.8%)	10 (10.2%)	8 (7.6%)	0.711
	Overall	18 (14.3%)	12 (9.5%)	17 (16.0%)	29 (21.6%)	0.058

Means compared using one way ANOVA; Medians compared using Kruskal Wallis test

Categorical variables compared using Chi Square or Fisher’s exact

*SD* Standard deviation, *IQR* interquartile range, *CS* Cesarean Section, *CSFD* Cesarean at full dilatation

*p* < 0.05 considered statistically significant

### Incidence of sPTB < 37, sPTB < 34 and second-trimester loss

**Fig. 2:**
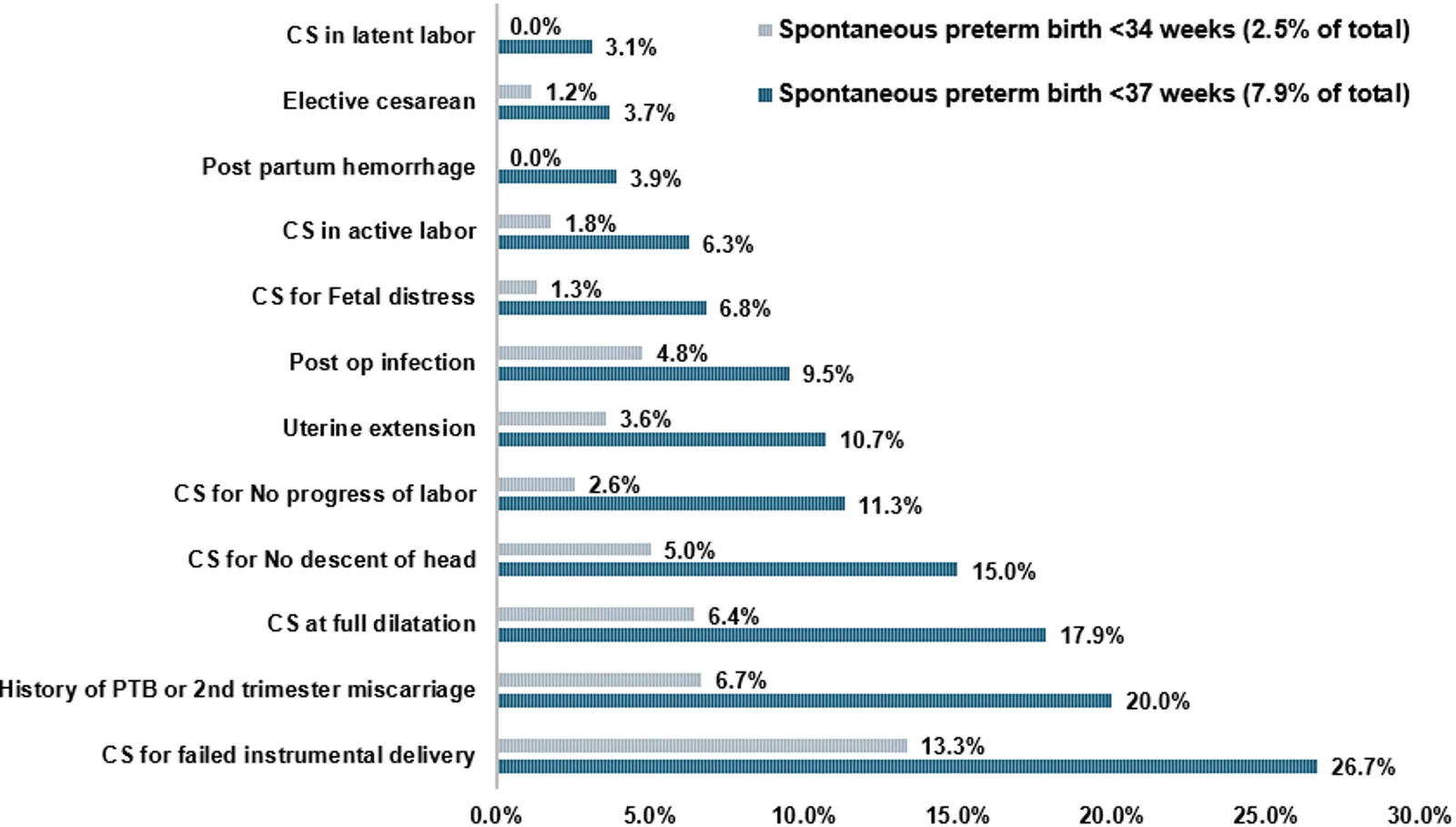
Proportion of women having spontaneous preterm birth < 34 weeks and < 37 weeks according to risk factors; CS- Cesarean section; PTB- preterm birth

**Fig. 3:**
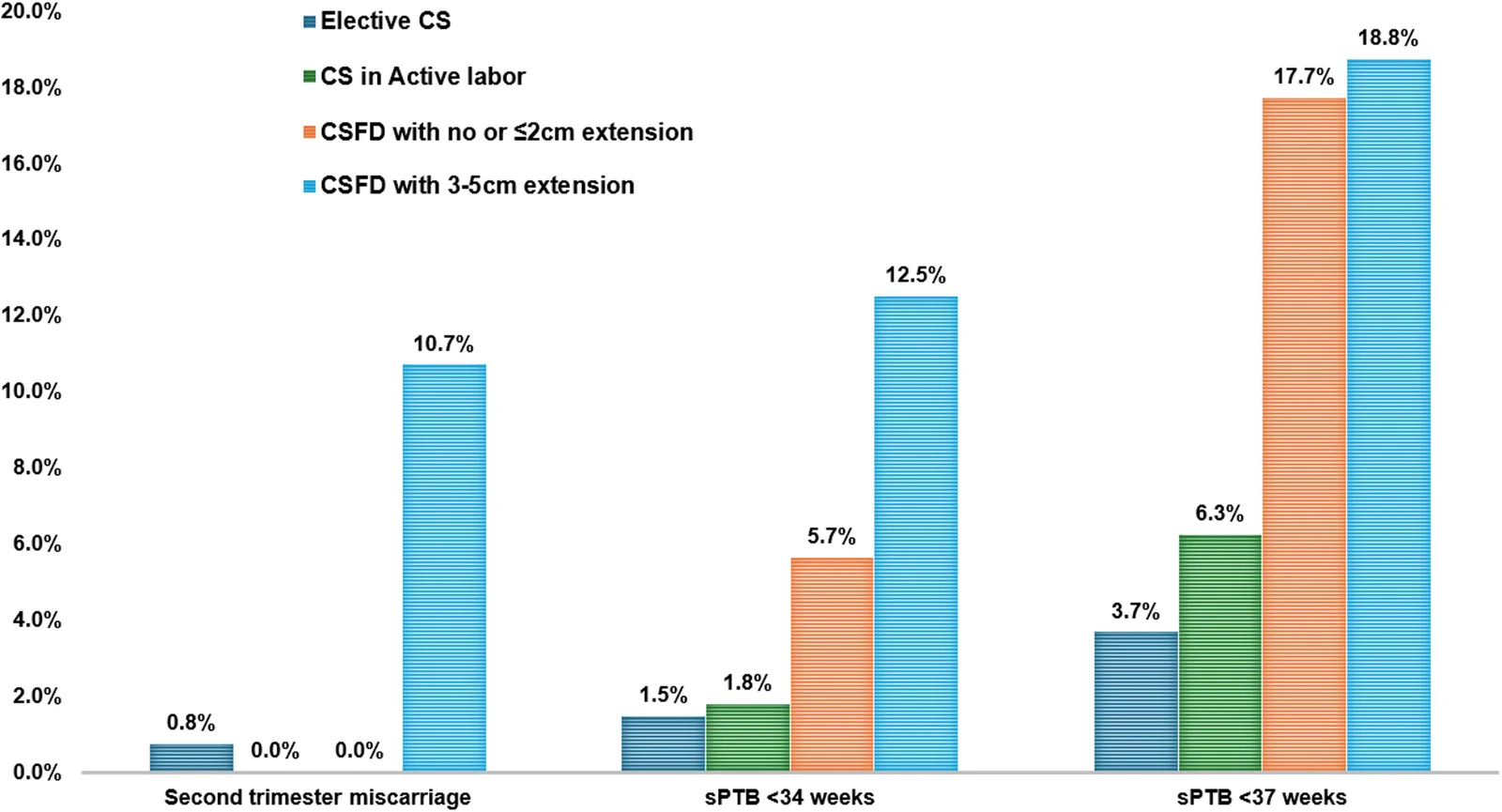
Pregnancy outcomes in women having CSFD with and without extensions; Numbers represent percentage in each group; sPTB- spontaneous preterm birth; CSFD- Cesarean at full dilatation; CS- Cesarean section

The primary outcome of PTB was evaluated in those having viable births (*N* = 530, missing data *N* = 13). Women in the CSFD group had significantly higher PTB < 34 weeks (7.9% in CSFD versus 2.2%, 0% and 1.8% in others, *p* < 0.001), and PTB < 37 weeks (22.1% in CSFD versus 7.4%, 6.2% and 7.1% in others, *p* < 0.001). The incidence of sPTB was 17.9% in CSFD, compared to 3–6% in others (*p* < 0.001). Among women with index pregnancy risk factors, 26.7% of those who had CS for FID had a subsequent sPTB (Fig. 2), higher than the 20% risk seen in those with history of PTB. CS for no progress of labour, post-op infections and uterine extensions also increased sPTB, compared to the overall cohort risk. The impact of extensions in CSFD, on pregnancy outcomes was explored as shown in Fig. 3. Strikingly, 10.7% of CSFD with 3–5 cm extension had a second-trimester miscarriage (< 1% in other groups) and 12.5% had a sPTB < 34 weeks (< 6% in other groups). However, extensions did not further increase the risk of sPTB < 37 weeks in those with CSFD.

### Adjusted risk ratios for sPTB < 37 weeks

**Fig. 4:**
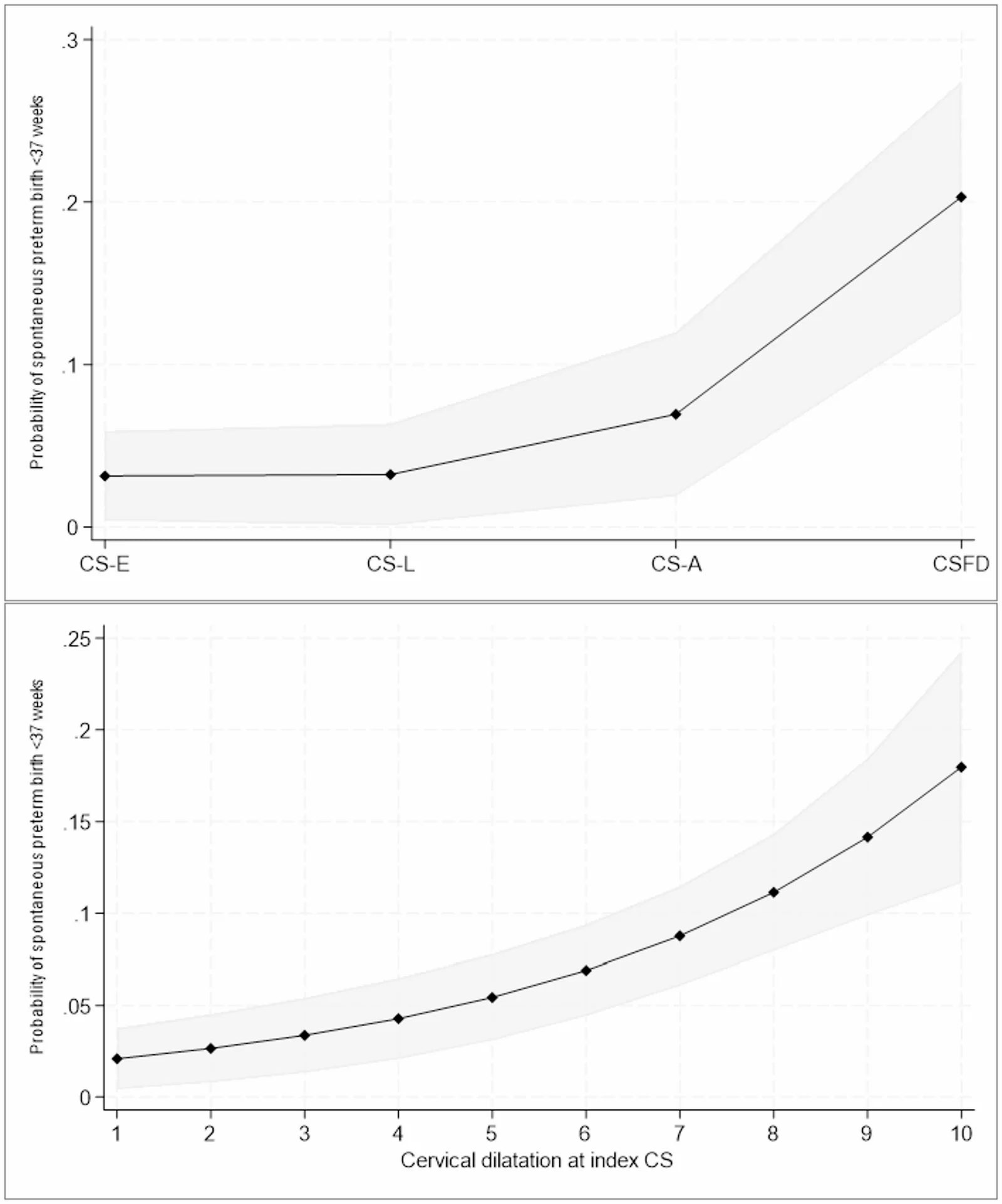
Predicted adjusted risks from GLM models for sPTB < 37 weeks; CS-E- Elective cesarean; CS-L- Cesarean section in latent phase of labour; CS-A- Cesarean section in active phase of labour; CSFD- Cesarean section as full dilatation

After adjusting for important confounders, the CSFD group had a 6.5 times higher risk of sPTB compared to CS-E (aRR = 6.47; CI = 2.47–16.9; *p* < 0.001). CS-A had more than twice the risk of sPTB than CS-E; however, this was not statistically significant (aRR = 2.21; CI = 0.71–6.91;*p* = 0.173). For every unit increase in cervical dilatation at the time of the index CS, risk of sPTB increased by 27% (aRR = 1.27; CI = 1.14–1.41;*p* < 0.001). CS for NDH and FID had 5 times (aRR = 5.32; CI = 2.20–12.8; *p* < 0.001) and nearly 9 times higher risk (aRR = 8.73; CI = 2.72-28.0; *p* < 0.001), respectively, compared to elective indications (Table 3). CSFD with large extensions increased the risk of sPTB more than 8 times (aRR = 8.18; CI = 2.27–29.5; *p* = 0.001). In the subgroup of women (*N* = 381) who had their first pregnancy in 2017, CSFD increased the risk of sPTB more than 3 times (aRR 3.29; CI = 1.13–9.59; *p* = 0.029), with every cm increase in cervical dilatation increasing the risk by 18% (CI = 1.05–1.33; *p* = 0.007). The predicted probabilities of sPTB are shown in Fig. 4.

**Table 3:** Risk ratios for sPTB < 37 weeks in the first subsequent pregnancy from binomial GLM models

Exposures in those with viable subsequent births		Unadjusted RR (95% CI)	*P* value	Adjusted RR (95% CI)	*P* value
Spontaneous preterm birth < 37 weeks in all women who had viable births					
*Model 1*: Groups (*N* = 507)	Elective CS	Ref	-	Ref	-
	CS in latent labour	0.84 (0.23–3.07)	0.796	1.03 (0.28–3.83)	0.967
	CS in active labour	1.70 (0.55–5.21)	0.353	2.21 (0.71–6.91)	0.173
	CS at full dilatation	4.86 (1.91–12.3)	0.001	6.47 (2.47–16.9)	< 0.001
*Model 2*: Cervical dilatation at CS (for every unit increase) (*N* = 507)		1.23 (1.11–1.37)	< 0.001	1.27 (1.14–1.41)	< 0.001
*Model 3*: Indication for index cesarean (*N* = 507)	Other indications	Ref	-	Ref	-
	No progress in labour	1.58 (0.52–4.84)	0.419	2.37 (0.75–7.52)	0.142
	Suspected fetal distress	1.64 (0.64–4.19)	0.305	2.18 (0.83–5.75)	0.115
	No descent of head at 10 cm	3.71 (1.56–8.78)	0.003	5.32 (2.20–12.8)	< 0.001
	Failed instrumental delivery	6.56 (2.17–19.9)	0.001	8.73 (2.72-28.0)	< 0.001
*Model 4*: CSFD with extension (*N* = 272)	Elective CS	Ref		Ref	
	CSFD (no/ ≤2 cm extension)	4.86 (1.88–12.4)	0.001	7.77 (2.76–21.8)	< 0.001
	CSFD (3–5 cm extension)	5.10 (1.34–19.4)	0.017	8.18 (2.27–29.5)	0.001
Spontaneous preterm birth < 37 weeks in women who were primigravida at the time of CS in 2017					
*Model 5*: Groups (*N* = 382)	Elective CS	Ref	-	Ref	-
	CS in latent labour	0.69 (0.16-3.00)	0.620	0.73 (0.17–3.21)	0.682
	CS in active labour	1.34 (0.37–4.82)	0.656	1.37 (0.38–4.98)	0.631
	CS at full dilatation	3.11 (1.08–8.99)	0.036	3.29 (1.13–9.59)	0.029
*Model 6*: Cervical dilatation at CS (for every unit increase) (*N* = 382)		1.18 (1.04–1.33)	0.009	1.18 (1.05–1.33)	0.007

All models adjusted for maternal age, parity, BMI, nationality, history of preterm birth or second trimester miscarriage and preexisting comorbidities

*CS* Cesarean section, *CSFD* Cesarean at full dilatation, *RR*-Risk ratios calculated using GLM models for binomial family for each exposure, 95% confidence intervals reported, *Ref* Reference or baseline group

### Further conceptions over the follow-up period (Supplementary T1)

More than 50% of women having a first conception (*N* = 343; 34.8% of total) had a second pregnancy after an additional median interpregnancy interval of approximately 2 years (Fig. 1). The incidence of first-trimester miscarriage in these pregnancies was 14.5%; 84% had a viable birth, among whom 9.7% had sPTB (11–12% in women who had CSFD and CS-A in 2017, compared to 9% in CS-E and 6% in CS-L (*p* = 0.635)). However, 66.7% of those who had a sPTB in the first conception had a PTB in the second. 12% of the cohort had a subsequent third conception (12.6% with sPTB, 80% recurrent PTB), and 4.6% had a subsequent fourth conception (9.4% with sPTB).

## Discussion

In this Middle Eastern cohort with a PTB rate of 8–9% in the population, women having CSFD had more than six times higher adjusted risk (absolute risk 17.9%) of having a sPTB in the subsequent pregnancy compared to those having an elective CS (absolute risk 3.7%). Having CSFD with extensions significantly increased the risk of second-trimester miscarriages (10%) and sPTB < 34 weeks (12.5%). Nearly 1/3rd of those having a CS for failed instrumental delivery had a subsequent sPTB.

### Findings in the context of what is known

The biological plausibility of an association between CSFD and sPTB stems from the fact that the cervix is completely taken up into the lower uterine segment in the second stage of labour, predisposing the operator to inadvertently injure cervical muscular tissue, which is instrumental in maintaining the integrity of the internal os in future pregnancies [19]. Many studies over the past decade have explored this possible etiology, consistently reporting that CSFD increases the risk of subsequent sPTB [11], although with varying strengths of association depending on the baseline sPTB rates. In 2014, Levine et.al [9] reported a six times higher odds of sPTB in CSFD compared to first-stage (USA national sPTB = 7–8%), including all women regardless of parity, which is consistent with the results in our study. A recent Danish study (2025) of nearly 400,000 nulliparous women (sPTB 1.7%) reported 40–50% increased odds of subsequent sPTB in CSFD compared to VB and first-stage CS [20]. In comparison, the RR was more than double in our nulliparous subgroup, possibly due to a larger PTB risk in the population.

A large Aberdeen study, including nulliparous women (sPTB 3–4%), reported more than 3 times higher adjusted odds of subsequent sPTB with CSFD compared to all other births [21], similar to our subgroup analysis. A 2023 study conducted in Bahrain, with similar demographic profile and sPTB rate as Qatar, reported a 23% risk of PTB in those with CSFD compared to 10% in the first-stage; however, there was no differentiation between spontaneous and iatrogenic PTB [22]. We report a similar 22% risk of overall PTB, but have focused our analysis on sPTB, which is more reflective of cervical integrity. Our cohort selection was similar to previous studies since we included women having a term CS, but on an unscarred uterus to eliminate the potential impact of multiple uterine scars on subsequent conceptions and births.

Few studies have compared CS-E and CS across different phases of the first-stage of labour. A 2020 study from Scotland reports a similar sPTB risk in those with VB, CS-E, and CS in latent and active first-stage, 3.3 times higher odds with CSFD compared to VB, and 15% increased odds for every unit increase in cervical dilatation [23]. However, they included nulliparous women and defined active labour from 4 cm cervical dilatation. Since we have included multiparous women as well, we decided to adopt the recent ACOG cut-off of 6 cm for active labour [16], possibly explaining the stronger association observed between CS-A and sPTB in our study. Additionally, we report a 27% higher risk with every cm dilatation, probably due to the higher PTB risk in our population. Williams et al. also report statistically significant associations between CSFD and sPTB < 34 weeks and consistently similar rates in CS-E and CS-L, which agree with our results. When repeating the analysis in our nulliparous subgroup, the RRs for sPTB are very similar to those reported by Williams et al., supporting the validity of our findings.

Nearly a fourth of CSFDs result in inadvertent extensions due to fragile, swollen vascular tissue following sustained pressure from the fetal head [6, 7]. In our cohort, more than one-fourth of CS for FID/NDH, 20% of CSFD and 12% of CS-A had extensions, very similar to the Canadian study by Muraca et al., exploring the effect of extensions on subsequent sPTB [8]. They reported a 40% increased risk of sPTB < 37 weeks with extensions, not reaching statistical significance. We encountered a similar issue due to smaller numbers but were able to replicate a similar trend. Additionally, we were able to specifically explore larger extensions (3–5 cm) and report an increasing trend. Muraca et al. reported a significant 2.4-fold higher risk of sPTB < 34 weeks. In our cohort, the impact of CSFD with large extensions was evident from the striking increase in sPTB < 34 weeks in that group, indicative of reduced cervical function and eventual incompetence. However, the inherent bias associated with subjective estimates of extensions should be kept in mind, and the impact is likely larger than what we have reported.

Interestingly, although CS for FID and NDH had similar rates of extensions, FID was the strongest risk factor for sPTB < 34 and < 37 weeks in our cohort, as previously reported [11]. This implies that apart from extensions, associated factors such as duration of second-stage, lower impacted fetal head, ascending infections, and maternal factors associated with both FID and sPTB might play a role. Although reviews report more than twice the risk of subsequent sPTB with CSFD compared to an instrumental VB [24], it must be borne in mind that apart from maternal factors, physician experience plays a key role in FID [25]. Our findings highlight the importance of clinical protocols and maintenance of instrumental VB skills to reduce FID and the associated surgical trauma, as well as the requirement for accurate descriptions of extensions to help with appropriate risk assessment of subsequent pregnancies.

The evidence regarding second-trimester miscarriages is still preliminary, as most registry-based studies have failed to report this outcome. Woolner et al. explored this outcome in 979 women having CSFD and reported no increased risk of miscarriages of any gestation when compared to VB or CS-E [26]. We observed no difference in miscarriages when comparing the main CS groups. However, there is a significant increase in second-trimester loss in CSFD with large extensions (Fig. 3). Although the numbers are small, this has not been demonstrated in other studies. Similarly, recurrent sPTB and second-trimester losses in further conceptions have been explored previously, with CSFD emerging as a significant risk [27, 28], studies demonstrating a high incidence of recurrent losses < 24 weeks in spite of having a vaginal cerclage [29]. We report similar risks of recurrent sPTB; however, there were no significant differences between groups due to small numbers.

### Clinical implications

This study adds valuable insight regarding the consequences of the surgical trauma associated with CSFD. It’s clear that CSFD is a risk factor for subsequent pregnancy losses or sPTBs and has been incorporated into current guidelines. The effective use of imaging, such as a transvaginal ultrasound [30] to determine CS scar niches as a marker of cervical integrity, may greatly improve prediction, in addition to standard cervical length screening. Preventive measures are particularly challenging in this group, as transabdominal cerclages may perform better than transvaginal due to better placement of stitch above the scar defect [29], and there is a lack of expertise for the same. In Qatar, CSFD may need to be incorporated into the risk assessment for sPTB, as the rate of CSFD in nulliparous women is increasing, resulting in an inevitable risk of subsequent sPTB. Gaining local expertise in advanced scar imaging and abdominal cerclages can prove beneficial in tackling this rising problem. Considering the use of vaginal progesterone from early pregnancy in this high-risk group might be warranted.

### Strengths and limitations

The strength of our study lies in the novel exploration of the impact of the degree of extensions on adverse outcomes. We considered all CS performed over 12 months, thereby reducing any temporal bias due to changing expertise or surgical and infection control protocols. The study was well-powered for the primary outcomes and was adequately sized to evaluate important exploratory associations. Adjusted analysis accounted for maternal factors and past obstetric history that are independent risk factors for sPTB.

However, certain limitations need to be kept in mind while analysing the results of this study. The analyses of the primary outcomes included women who subsequently conceived, essentially excluding those who were unable to and those who chose not to. It’s possible that women with more serious pathology were excluded. However, the ability to conceive was not one of our outcomes, and the cohort we focused on was clinically more relevant to high-risk obstetric practice. The retrospective nature of the study can result in residual confounding due to unaccounted-for variables. We did not include a proxy for socio-economic status; however, nationality was included in the model to account for potential ethnic variations. There are several potential in-labour factors, such as duration of first or second stage and use of oxytocin, that reflect labor complexity and contribute to a difficult CS. Since these factors have not been independently linked to future PTB, we have not considered them as confounders. Most variables (especially exposure and outcomes) are unlikely to be misclassified; however, human error during data processing cannot be eliminated.

Documentation of uterine extensions is always subject to inter-surgeon bias, as an underestimation of the size is the norm, resulting in a possible underestimation of the impact. Hence, the findings related to uterine extensions are exploratory and warrant further evaluation in larger cohorts. A complete-case analysis was performed, excluding the women who chose to deliver in a different setting; however, these numbers were small (2.5%, < 1% in CSFD) and unlikely to affect the results. Although cervical injury during CS is a compelling, biologically plausible risk factor for future sPTB, the precise causal pathways are not completely understood, and other contributing factors, such as inflammation, labor factors, infection, uterine scar remodelling, etc., may contribute.

## Conclusion

CSFD is associated with a 6.5-fold higher risk of sPTB compared to elective CS. Indications for the CS (especially FID) and uterine extensions are other important potential predictors for sPTB and second-trimester miscarriage. Risk assessment for PTB might need to include detailed CS history and review of operative records. Women with CSFD might potentially require referral to dedicated PTB clinics and be offered preventive measures such as vaginal progesterone and serial cervical length assessments. Future prospects will involve gaining expertise in innovative predictive techniques, such as ultrasound-based scar niche assessment, and in preventive surgical procedures, including abdominal cerclage.

## Supplementary Information

Supplementary Material 1.

## Data Availability

Deidentified data that support the findings of this study are available upon reasonable request from the corresponding author (fminisha@hamad.qa). The data are not publicly available due to privacy or ethical restrictions. In order to access the data, a formal request specifying the reason for the request and how the data will be used, must be sent to the corresponding author. Availability is subject to approval from the Medical Research Centre, Hamad Medical Corporation, Qatar.
