# Supplementary material for: Risk of preterm birth and second-trimester loss following intrapartum cesarean sections and uterine extensions: a population-based cohort study
Source: Reprod Health. 2026 Jun 19;23:209. doi: 10.1186/s12978-026-02387-w (PMC13753319; doi:10.1186/s12978-026-02387-w)
Supplement: Supplementary file 1 [file 12978_2026_2387_MOESM1_ESM.docx]

| Supplementary Table 1- Outcomes of second and third subsequent pregnancies | | | | | | | |
| --- | --- | --- | --- | --- | --- | --- | --- |
| Outcomes of the first subsequent pregnancy | Conception  n (%N) | First-trimester miscarriage | Second-trimester miscarriage | Viable births ≥24 weeks | sPTB<34 weeks | sPTB<37 weeks | PTB <37 weeks in those with sPTB in prior conception |
| **Second conception (all groups)** | **343 (53.9%)** | **48 (14.5%)** | **5 (1.5%)** | **278 (84.0%)** | **7 (2.5%)** | **27 (9.7%)** | **14 (66.7%)** |
| Elective CS (N=162, missing=3) | 84 (51.9%) | 15 (18.5%) | 2 (2.5%) | 64 (79.0%) | 2 (3.1%) | 6 (9.4%) | 2 (50.0%) |
| CS in latent labour (N=161, missing=1) | 84 (52.5%) | 16 (19.3%) | 0 (0%) | 67 (80.7%) | 2 (3.0%) | 4 (6.0%) | 1 (100.0%) |
| CS in active labour (N=145, missing=4) | 79 (54.5%) | 7 (9.3%) | 2 (2.7%) | 66 (88.0%) | 3 (4.6%) | 8 (12.1%) | 2 (50.0%) |
| CSFD (N=168, missing=4) | 96 (56.8%) | 10 (10.9%) | 1 (1.1%) | 81 (88.0%) | 0 (0%) | 9 (11.1%) | 9 (75.0%) |
| **Third conception (all groups)** | **119 (34.7%)** | **21 (18.1%)** | **3 (2.6%)** | **95 (81.9%)** | **4 (4.2%)** | **12 (12.6%)** | **4 (80.0%)** |
| Elective CS (N=84, missing 2) | 30 (35.7%) | 3 (10.7%) | 1 (3.6%) | 26 (92.9%) | 1 (3.8%) | 2 (7.7%) | 1 (100%) |
| CS in latent labour (N=84) | 34 (40.5%) | 7 (17.6%) | 0 (0%) | 27 (79.4%) | 1 (3.7%) | 5 (18.5%) | 1 (100.0%) |
| CS in active labour (N=79) | 22 (27.8%) | 6 (27.3%) | 1 (4.5%) | 15 (68.2%) | 0 (0%) | 1 (6.7%) | 0 (0%) |
| CSFD (N=96, missing 1) | 33 (34.4%) | 5 (15.6%) | 1 (3.1%) | 27 (84.4%) | 2 (7.4%) | 4 (14.8%) | 2 (66.7%) |
| CS- Cesarean section; CSFD- Cesarean section at full dilatation; sPTB- spontaneous preterm birth; PTB- all preterm birth | | | | | | | |
